# Minocycline Modulates Human Social Decision-Making: Possible Impact of Microglia on Personality-Oriented Social Behaviors

**DOI:** 10.1371/journal.pone.0040461

**Published:** 2012-07-13

**Authors:** Takahiro A. Kato, Motoki Watabe, Sho Tsuboi, Katsuhiko Ishikawa, Kazuhide Hashiya, Akira Monji, Hideo Utsumi, Shigenobu Kanba

**Affiliations:** 1 Department of Neuropsychiatry, Graduate School of Medical Sciences, Kyushu University, Fukuoka, Japan; 2 Innovation Center for Medical Redox Navigation, Kyushu University, Fukuoka, Japan; 3 Graduate School of Economics, Waseda University, Waseda, Japan; 4 Department of Psychology, Graduate School of Letters, Kyoto University, Kyoto, Japan; 5 Graduate School of Human-Environment Studies, Kyushu University, Fukuoka, Japan; George Mason University/Krasnow Institute for Advanced Study, United States of America

## Abstract

**Background:**

Microglia, one of the glial cells, play important roles in various brain pathologies including psychiatric disorders. In addition, microglia have recently been proved to monitor synaptic reactions via direct-touching even in normal brain. Human microglia may modulate various social/mental functions, while microglial social/mental roles remain unresolved especially in healthy humans. There is no known drug with the specific effect of modulating microglia. Therefore, using minocycline, a tetracycline antibiotic and the most famous microglial inhibitor, is one of the best alternative approaches to clarify microglial functions on human social/mental activities.

**Methodology/Principal Findings:**

We conducted a double-blind randomized trial of trust game, a monetary decision-making experiment, with ninety-nine human adult males who decided how much to trust an anonymous partner after a four-day administration of minocycline. Our previous pilot trial indicated a positive effect of minocycline, while the underlying mechanisms were not clarified. Therefore, in this trial with larger samples, we additionally measured the effects of anxiety and personality. The monetary score in trust game was significantly lower in the minocycline group. Interestingly, participants’ ways of decision-making were significantly shifted; cooperativeness, one component of personality, proved to be the main modulating factor of decision-making in the placebo group, on the other hand, the minocycline group was mainly modulated by state anxiety and trustworthiness.

**Conclusions/Significance:**

Our results suggest that minocycline led to more situation-oriented decision-making, possibly by suppressing the effects of personality traits, and furthermore that personality and social behaviors might be modulated by microglia. Early-life events may activate human microglia, establish a certain neuro-synaptic connection, and this formation may determine each human’s personality and personality- oriented social behaviors in later life. To explore these mechanisms, further translational research is needed.

**Trial Registration:**

UMIN clinical trial center UMIN000004803

## Introduction

Microglia are one of the glial cells with immunological/inflammatory functions, and contribute to various brain pathologies; not only in neurodegenerative diseases [1], [2], [3] but also in psychiatric disorders such as schizophrenia and autism [4], [5], [6]. Minocycline, a tetracycline antibiotic, is known as the most famous microglial inhibitor [7], which has recently been applied to brain diseases such as stroke and neurodegenerative diseases [8], [9]. In addition, minocycline has been suggested to be an effective drug for psychiatric disorders [10], [11]. These reports suggest that inhibiting microglial activation may modulate human social and mental activities, and rodent studies have indicated this possibility [12], [13].

Rodent microglia have recently been shown to monitor synaptic reactions via direct-touching not only in pathological brain but also in normal brain [14], [15], [16], [17], and have proved to play important roles in normal brain development such as synaptic pruning [18]. Neurons and neuronal networks including synapses have been dominantly believed to play crucial roles in human social/mental activities. The above-mentioned evidence indicates that human microglia may modulate various social/mental functions, while microglial social/mental roles continue to remain unresolved especially in healthy humans.

There is no known drug with the specific effect of modulating microglia. Therefore, using minocycline, a tetracycline antibiotic and the most famous microglial inhibitor, is one of the best alternative approaches to clarify microglial functions on human social/mental activities. One human study suggests that minocycline attenuates the subjective reward effects of dextroamphetamine [19], while, to our knowledge, the effects of minocycline on human social/mental activities are not well understood.

Crockett et al have revealed that serotonin modulates behavioral reactions to unfairness, via a monetary decision-making game with healthy volunteers who were administered tryptophan-depleted amino acid which induces lower serotonin levels [20]. In order to measure human social/mental activities, these monetary decision-making experiments have been actively applied because such experiments enable the analysis of the interaction between social/mental activities and actual social behaviors [21], [22]. Pharmacology-based neuro-economic research is showing that human social behaviors are modulated by neurotransmitters such as serotonin and oxytocin [20], [23], [24], [25]. In addition, a significant link has recently been reported between the dopamine D4 receptor gene and fairness preference in ultimatum game [26]. However, the pharmacological interaction of social decision-making beyond neurotransmitters remains to be clarified [27].

As a first step in this direction, we recently conducted a pilot experiment with trust game, one of the decision-making experiments, with minocycline [28]. The forty-nine participants, healthy adult humans, made a monetary decision about whether or not to trust an anonymous partner after a four-day oral administration of minocycline or placebo. The minocycline group showed a strong and positive correlation between their scores in trust game and their pre-evaluation scores in others’ trustworthiness, but the placebo group did not. These pilot data have suggested that inhibitory effects of microglial activation may sharpen a sense of trust in social behavior, and this effect would enhance situation- oriented behaviors according to immediate social situations. In trust game, a player’s optimal decision depends on his/her prediction about the other player’s decision. Thus, social environment, including the other’s behavior, determines what behavior the player should take. In our actual life, however, our decisions are determined not only by social environment but also by our fundamental mental factors such as temperament and character (i.e. personality), which are independent from situational factors and may strongly impact on decision-making. These factors may act as a “noise” in trust game and during similar human decision-making situations [29]. Our pilot data demonstrated that only the minocycline group showed situation- oriented decision-making, which suggests that microglia may be inducing the “noise” consistently and inhibiting microglial activation could reduce this “noise” effect. However, the underlying mechanisms of “noise” were not clarified in our previous trial [28], thus the next appropriate step is the measurement of the effects of not only trustworthiness but also anxiety and personality.

Therefore, to clarify the microglial “noise” effect during human social decision-making, we newly explored whether anxiety and personality as a “noise” influences outcomes of trust game on humans with minocycline or placebo. To improve the small sample size and the weaker statistical power of our previous trial, we newly conducted the trial with larger samples (about one hundred participants).

## Methods and Materials

**Figure 1:**
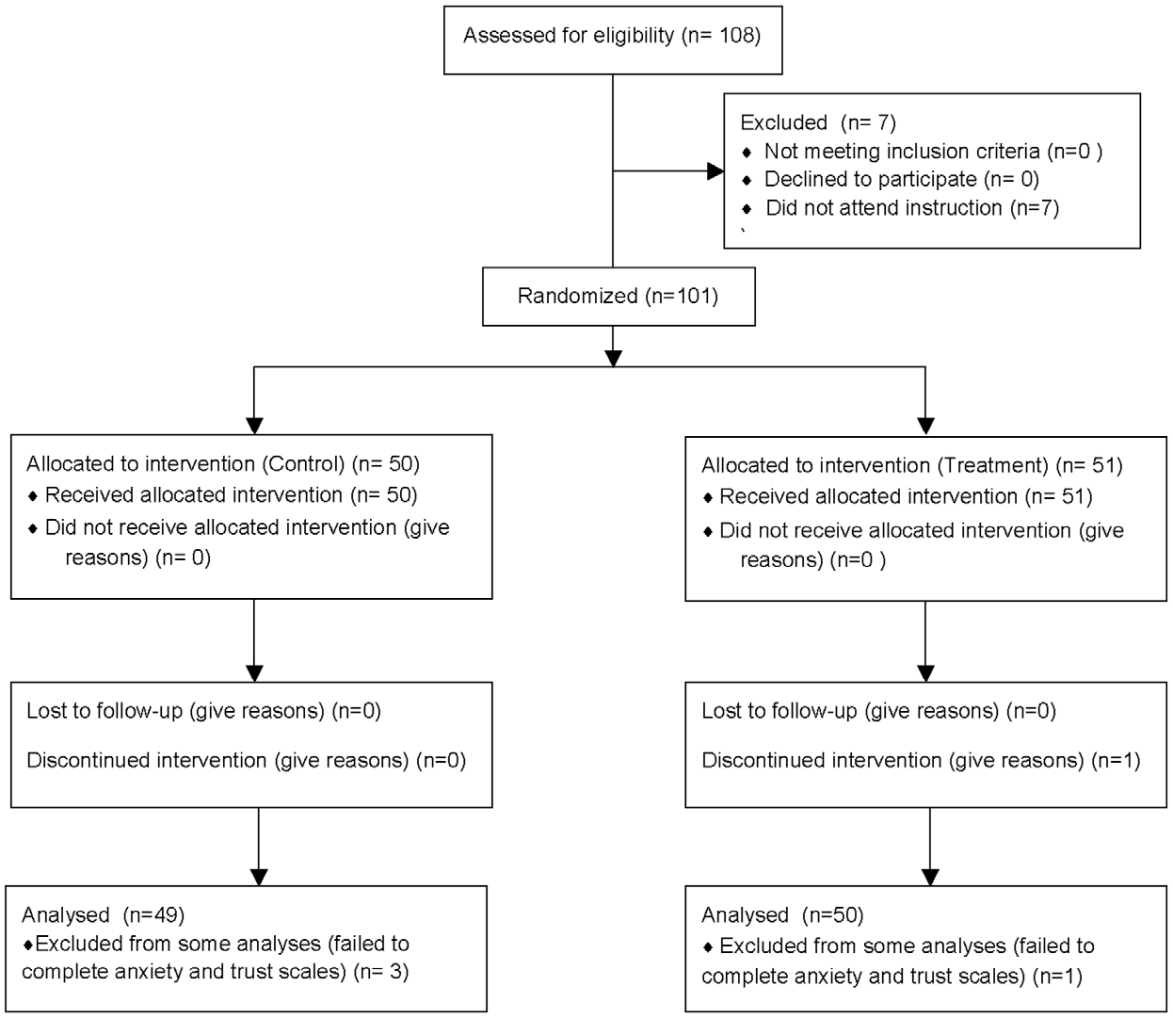
Flow Diagram of This Study.

The protocol for this trial and supporting CONSORT checklist are available as supporting information; see Checklist S1, Protocol S1 and Protocol S2. This double-blind randomized study was approved by the Kyushu University Ethical Committee under the administration of the UMIN clinical trial center (**UMIN000004803**). All the participants of the present experiment, which was conducted in December 2010, were unique to this study and differ from the previous participants who enrolled in an earlier experiment in March 2010 under the administration of the UMIN clinical trial center (UMIN000003281: published in Watabe et al. Psychopharmacology 2012). Flow diagram of this study is listed on **Figure 1**. All participants gave written informed consent to participate after a complete description of the study. Participants were administered minocycline or placebo for four days, after which they played a trust game with an anonymous partner.

### Subjects

Participants were recruited by advertisements on campus. Inclusion criterion was as follows; healthy adult males from 20 to 30 years old who can obtain informed consent. Exclusion criteria were the following five items; 1) those who have had side effects to antibiotics including minocycline, 2) those who have severe heart, liver or kidney disease, 3) those who have a tendency to develop allergies, and 4) those who have been diagnosed with psychiatric disorders. Their mental and physical health was confirmed by interview with a psychiatrist (TAK). All the participants were qualified for this study (**Table S1**).

### Drug Administration

Participants received a sheet describing their detailed dosing schedule. They were then asked to write the exact time of every dosing, and to submit every capsule package as evidence of dosing. Participants started to take a capsule in the evening of the first day and twice daily (morning and evening) for four days afterward. On the day of the game experiment (the fifth day), they were instructed to take the last capsule three-hours prior to the appointment time for the experiment so that all participants played the trust game under the similar drug effect. Each capsule contained 100 mg minocycline (in the treatment group) or 100 mg lactose (in the placebo group). This minocycline dose (200 mg/day) is within the range of the usual daily dose used for treatment of infections [30], and this dose has also been applied in recent clinical trials [10], [19]. Participants were randomly assigned to the treatment group or to the placebo group in advance, with a double-blind procedure.

### Procedure

Prior to drug administration, participants completed a set of questionnaires (details in **Scales**). After four days of drug administration, participants were interviewed by physicians regarding side effects, other medications, and adherence to the drug administration protocol. They then played a trust game [21] and responded to the same set of questionnaires they had completed before administration.

### Trust Game

**Figure 2:**
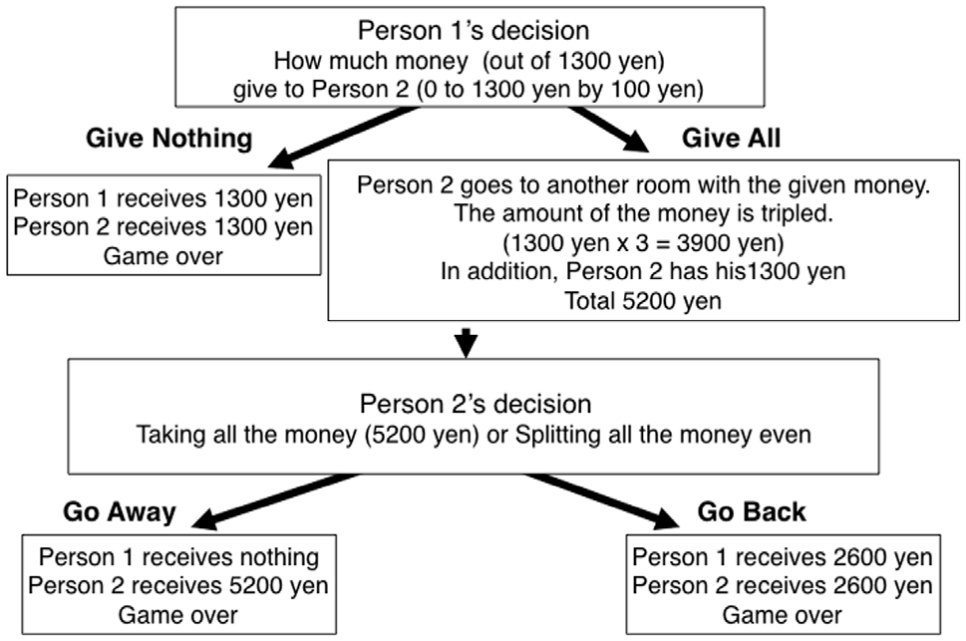
Trust Game Structure.

**Figure 2** shows the structure of trust game. In this two-player game, each player was initially given 1300 JPY (nine hundred JPY had been used in our previous trial [28], but to let participants recognized clearer incentive and make their decisions more seriously, we used 1300 JPY (about 15 USD) in this new trial so that we can obtain more reliable behavioral data). The first player then decided how much of the 1300 JPY to give to the second player. The second player then went to another room, where the amount of money given to him by the first player was tripled. The second player then decided whether to split his money equally with the first player or to take all of his money. In this experiment, all the participants were actually assigned to be the first player. The first player’s decision as to how much money to give to the second player is thought to be the first player’s level of trust in his partner. The amount of money given was expected to be a behavioral measure of the first player’s trustfulness.

In this experiment, participants had no information about the partner except that he was male. The participants thus were likely to have made their decisions based primarily on how much they trusted others in general. All the participants’ partner was actually a research confederate and always the same person, a 22-year-old Japanese male. In order to control the participant’s impression of the partner, the partner acted and talked exactly in the same way throughout all the experimental sessions.

### Scales

Our previous trial showed the positive correlation between participants’ scores in trust game and their pre-evaluation scores in others’ trustworthiness, while we did not examined other psychological factors and thus the underlying mechanisms were not clarified [28]. Therefore, we examined the effects of anxiety and personality, in addition to the trust scores, in this trial. The following self-rated questionnaires were completed by the participants at pre- and post-treatment.

### Temperament and Character Inventory (TCI)

TCI is based on the seven-factor model of temperament and character in personality [31]. According to TCI model, personality is classified into temperament, which consists of Novelty Seeking (NS), Harm Avoidance (HA), Reward Dependence (RD), Persistence (PS), and character, which consists of Self-Directedness (SD), Cooperativeness (C), Self-Transcendence (ST) with a four-point Likert type scale. We used a Japanese version with 125 questions (TCI-125) [32], which was kindly provided for use in this study from the HUMAN CAPITAL CONSULTING Corporation, Tokyo, Japan.

### State-Trait Anxiety Inventory (STAI)

This anxiety scale with 20 questions consists of two factors; state anxiety, which refers to relatively unstable emotional threat to current situations, and trait anxiety, which refers to relatively stable emotional threat consistently felt in daily life [33].

### General Trust Scale (GTS)

GTS consists of six questions with a seven-point Likert type scale developed by Yamagishi and Yamagishi [34]. This scale measures respondents’ estimation of others’ trustworthiness. The reliability and validity of GTS have been confirmed across many countries [35]. According to past research on GTS, the major confounders of general trust are culture, sex and education level [34]. To eliminate the effects of these confounders, we recruited a homogenous sample as possible. As a result, all the participants were Japanese males and who had collage/university level educations so that we could test the effect of general trust without these confounders.

### Data Analysis

Ninety-nine Japanese males, out of 101 entries, completed our experiments (mean age 21.52 years, SD 1.65 years), and analysis was conducted on this data. Among the participants, four (three in the minocycline condition, one in the control condition) failed to complete the questionnaires of STAI and GTS, so the analyses including these two scales were performed with the data of the 95 participants. All of the data analyses were performed with SPSS ver.19.

## Results

In our previous trial, the statistical power was 0.766, and the statistical power in the present trial is 0.847. Therefore, the present trial exceeds the suggested efficient power of 0.8. The following analyses are shown with this more appropriate power.

### Behavior in Trust Game

We compared the mean amount of participants’ monetary offers in trust game by a t-test (**Table 1**). The monetary score in trust game was significantly lower in the minocycline group compared to the placebo group (*t*(97) = 2.08, *p*<.05). This result is consistent with our pilot study [28].

**Table 1:** Behavior in Trust Game, and Effects of Minocycline on Personality, Anxiety and Trust.

Category	Subcategory	Before Treatment		After Treatment		Before-After	Control-Minocycline	Interaciton
		Control	Minocycline	Control	Minocycline			
**Monetary Score in Trust Game (%)**	**–**	N/A	N/A	61.38 (32.43)	48.77 (27.70)	N/A	*t*(97) = 2.08, *p*<.05	N/A
**TCI (from 1 to 20)**	**Self-Transcendence**	10.69 (2.05)	10.82 (2.19)	10.52 (2.47)	10.57 (2.63)	*ns.*	*ns.*	*ns.*
	**Cooperative-ness**	14.98 (1.79)	14.98 (1.89)	14.80 (1.91)	14.79 (1.98)	*ns.*	*ns.*	*ns.*
	**Self-Directedness**	12.87 (0.20)	12.71 (0.22)	12.62 (1.98)	12.22 (2.09)	*ns.*	*ns.*	*ns.*
	**Persistence**	12.86 (2.14)	13.54 (2.53)	13.31 (2.34)	13.76 (2.25)	*ns.*	*ns.*	*ns.*
	**Reward Dependence**	14.07 (1.88)	14.28 (2.38)	14.10 (1.75)	14.10 (2.21)	*ns.*	*ns.*	*ns.*
	**Harm Avoidance**	13.51 (2.47)	13.30 (2.37)	13.55 (2.50)	13.26 (2.56)	*ns.*	*ns.*	*ns.*
	**Novelty Seekng**	12.97 (1.69)	12.99 (1.76)	12.94 (1.77)	13.02 (1.62)	*ns.*	*ns.*	*ns.*
**STAI (from 1 to 4)**	**State Anxiety**	2.04 (0.45)	2.00 (0.53)	2.11 (0.51)	2.28 (0.57)	*F*(1, 93) = 18.60 *p*<.01	*ns.*	*F*(1, 93) = 6.57 *p*<.05
	**Trait Anxiety**	2.33 (0.52)	2.21 (0.56)	2.27 (0.51)	2.27 (0.58)	*ns.*	ns.	ns.
**General Trust Score (from 1 to 7)**	**-**	4.31 (1.06)	4.51 (1.12)	4.41 (1.07)	4.53 (1.04)	*ns.*	*ns.*	*ns.*

We performed t-test on the behavior (monetary score) in trust game, and the average scores are shown in the Table. The effects of minocycline on personality, anxiety and trust were evaluated with the seven subscales of TCI, the two subscales of STAI, and GTS. We performed ANOVA with a repeated measure; the scores of the subscales as the dependent variable, and drug condition (Minocycline vs. Control), repeated measure of the subscales’ scores (*Before* vs. *After* treatment) and their interaction as independent variables. As four participants (three for control, one for minocycline group) failed to complete the questions of STAI and GTS, 95 sets of data were analyzed. Significant and/or marginal effects are shown in the Table. Results were expressed as means (S.D.).

### Effects of Minocycline on Personality, Anxiety and Trust

The effects of minocycline on personality, anxiety and trust were evaluated with the seven subscales of TCI, the two subscales of STAI, and GTS. We performed ANOVA with a repeated measure; the scores of the subscales as the dependent variable, and drug condition (Minocycline vs. Control), repeated measure of the subscales’ scores (*Before* vs. *After* treatment) and their interaction as independent variables (**Table 1**).

There was no significant interaction term on each of the subscale of TCI. The main effect of time (*Before* vs. *After* treatment) was significant for *Persistence*. The score of *Persistence* is higher *After* (mean score = 13.09, SD = .237) than *Before* (mean score = 13.46, SD = .236). No effect was found on the rest of the items. These results indicate that participants’ personality itself was not significantly affected by minocycline.

On STAI, interaction effect and main effect were significant on *Before-After* for state anxiety. Compared to the control group, the state anxiety score increased steeply in the minocycline group. According to simple main effect test, the score after the treatment was significantly higher in the minocycline group than in the control group (*p*<.001). Thus, this result may explain the cause of the lower trusting behavior for minocycline group in trust game. We found no significant effect on trait anxiety.

On GTS, there were no main or interaction effects.

### Effects of Minocycline on Decision-Making Style

Next, to examine the effects of minocycline on decision-making style, we performed a multiple linear regression analysis of the amount of money offered (monetary score) in trust game as the dependent variable, and subscales of TCI, STAI and GTS as independent variables by conditions (**Table 2**). We revealed that state anxiety (*β* = −.795, *t* = −4.42, *p* = .001) and trust (*β* = .321, *t* = 2.35, *p* = .023) have significant effects in the minocycline group (*R^2^* = .288, *F*(3,46) = 9.30, *p* = .001) while only cooperation scale of TCI (*β* = .486, *t* = 2.58, *p* = .013) was significant in the control group (*R^2^* = .092, *F*(3,42) = 2.51, *p* = .078). Our novel finding in the present study is that the effect of state anxiety was stronger than that of trustworthiness. In sum, for the minocycline group, the less state anxiety and the more trustful, the more trusting behavior; while for the control group, the more cooperativeness, the more trusting behavior.

**Table 2:** Multiple Regression Analysis on Behavior in Trust Game.

Independent Variable	Control Group	Minocycline Group
	Beta	Beta
Cooperativeness (TCI)	.486*	
Reward Dependence(TCI)	−.281	
Self-Directedness (TCI)	−.284	
State Anxiety (STAI)		−.583**
General Trust		.321*
	*N = 46, R^2^ = .092, F(3, 42) = 2.51, p<.10*	*N = 49, R^2^ = .288, F(2, 46) = 9.30, p<.001*

Note: *p<.05,

** p<.01.

We performed a multiple linear regression analysis of the amount of money offered in trust game as the dependent variable, and subscales of TCI, STAI and GTS as independent variables by conditions. Remarkable effects are shown in the Table.

## Discussion

As a first step to explore how microglia modulates human social/mental activities, we showed the novel effect of minocycline, the most famous inhibitor of microglial activation, on human monetary decision-making in trust game. Our previous trial, with smaller sample size and weaker statistical power, indicated the positive effect of minocycline on trust game, while the significant results were limited. In the present trial, we newly revealed that the monetary score in trust game was significantly lower in the minocycline group. Another novel finding was that minocycline treatment itself did not change personality, while, surprisingly participants’ ways of decision-making were significantly shifted; cooperativeness, one component of personality, was the main modulating factor of decision-making in the placebo group, on the other hand, the minocycline group was mainly modulated by state anxiety and trustworthiness, both of which are known to be mainly dependent on real-time environments such as present social situation. In addition, the effect of state anxiety was stronger than that of trustworthiness. These results suggest that minocycline led to more situation-oriented decision-making, supporting our “noise reduction” hypothesis [28]; participants’ personality may act as a “noise” during human social decision-making and minocycline may mimic personality- oriented behaviors.

### Impact of Microglia on Personality-Oriented Social Behaviors

**Figure 3:**
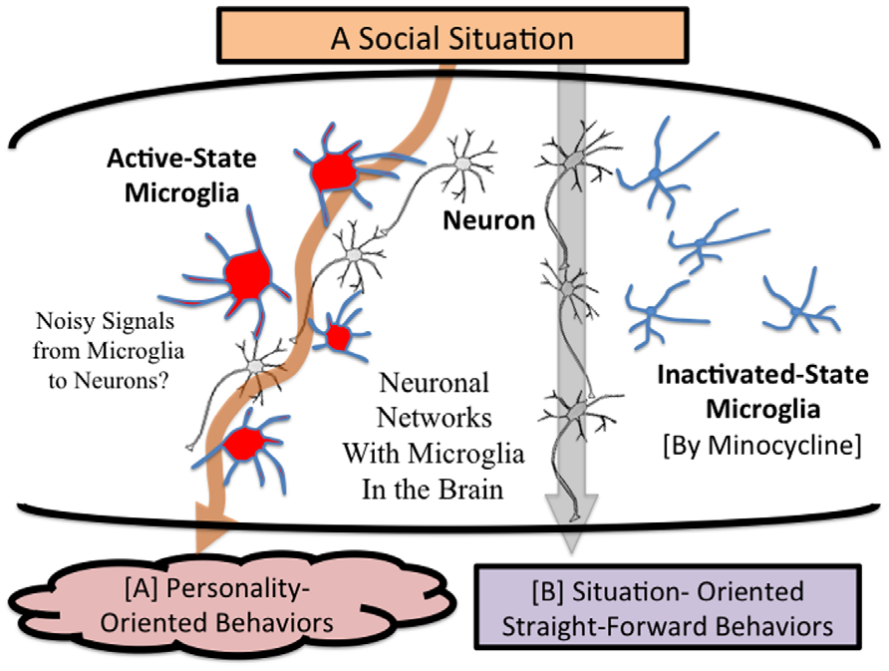
Possible Impact of Microglia on Personality and Social Behaviors. Early-life environmental experiences such as psychological stress and traumatic events may activate human microglia, establish a certain neuro-synaptic-microglial connection, which is memorized unconsciously as a primer for an extended period, and this formation in the human brain determines each human’s personality and personality- oriented social behaviors in later life. In sum, neuronal networks with active microglia may induce noisy-decision-making, which is equivalent to personality- oriented behaviors (A). On the other hand, decision-making with neuronal dominant networks may induce straightforward behaviors, which are less affected by personality (B). In the present study, the control group’s personality- oriented behaviors could be formulated by microglial priming effects (A), and the minocycline group’s situation- oriented behaviors may be induced by suppressing microglial contribution to social behaviors (B).

The novel effects of minocycline may explain the unknown role of microglia in social/mental activities. Until now, no study has reported microglial activities in healthy human subjects, while microglia have proved to play important roles in normal brain by communicating with neurons via releasing mediators and synaptic direct contact in rodent studies [14], [15], [16]. Therefore, human microglia may perform actively even in healthy brains, and inhibiting microglial activation with minocycline may create a shift from personality-oriented behaviors to situation-oriented behaviors by modulating neuro-synaptic-microglial networks. Rodent microglia play essential roles in synaptic pruning [18], which has pointed to the cryptic roles of microglia in human brain development. A recent study suggests that rodent microglial activation by infections during early developmental periods last, and these pre-activated microglia will be re-activated rapidly compared to normal state microglia [36]. Another study has suggested that microglia have a crucial role in the process of early-life memory in rats [37]. Early-life events can significantly modulate normal learning-dependent cytokine activity within the hippocampus, via a specific, enduring impact on brain microglial function, and preventing microglial activation by minocycline during learning prevents memory impairment in neonatally infected rats. Microglia are known to be activated not only by infection but also by physical and psychological stress in rodent studies [12], [38], [39], [40]. In addition, Wei et al. reported that early life stress inhibits expression of a novel innate immune pathway in the developing hippocampus in pups [41]. Based on these recent findings, we suggest the possible existence of the following mechanism on personality and social behaviors; early-life environmental experiences such as psychological stress and traumatic events may activate human microglia, establish a certain neuro-synaptic-microglial connection, which may be memorized unconsciously as a primer for an extended period, and this formation in the human brain may determine each human’s personality and personality- oriented social behaviors in later life (**Figure 3**). In addition, we can interpret the present results as follows; the control group’s personality- oriented behaviors could be formulated by microglial priming effects, and the minocycline group’s situation- oriented behaviors may be induced by suppressing microglial contribution to social behaviors. Further studies are needed to clarify contributions of microglia to human development including personality formation, and social/mental activities in later life.

### Clinical Implication

Minocycline has been suggested to be an effective drug for psychiatric disorders [10], [11]. Disturbed decision-making is a common symptom of various psychiatric disorders [42], [43], and this disturbance is treated by psychotropic drugs such as antipsychotics and antidepressants, which have proved to inhibit microglial activation from *in vitro* studies [44], [45], [46], [47], [48]. In addition, a recent study suggests that effort-based decision-making in rat is modulated by estradiol [49], a sex hormone, which also has inhibitory effects on microglial activation [50], [51]. These data support our minocycline results, and indicate that psychiatric treatments may modulate microglial contribution to disturbed decision-making in social behaviors. To develop our results and these perspectives, animal based decision-making experiments with minocycline (or other microglial inhibitors) and histological analysis of microglia are called for in the near future. In addition, clinical trials of social decision-making experiments focusing on microglia should be attempted.

### Limitation

First, this study did not examine the dose-dependent effects of minocycline. Second, this study was conducted only with adult males, while there may be a difference when players are female. Third, we did not measure microglia activity in the brain via imaging methods, while minocycline may inhibit some brain regional activities which are thought to be linked to trust and social decision-making [52], [53]. Therefore, brain imaging studies are needed to clarify these regional activation mechanisms. Finally, other possible minocycline effects should be taken into account. Apart from inhibiting microglial activation, minocycline also has been reported to interact with brain glutamate and dopamine neurotransmission [54], [55] and to have direct effects on neuronal cell line, PC12 [56]. Some reports suggest positive links between microglia, glutamate and dopamine interaction [57], [58]. Further research should be performed to clarify this interaction mechanism. No specific inhibitor of microglia exists to date, therefore we selected minocycline as the most appropriate and safest drug to be used in humans at present. When a safe, specific inhibitor of microglial activation is developed, microglial human function will be clarified more effectively.

### Conclusion

Based on the results of the present human social decision-making experiment, we have proposed a novel microglial contribution to personality and social behaviors. Our present study may shed new light on microglial roles in the social and mental life of healthy humans and also of people with psychiatric disorders. To explore these perspectives, further *in vitro/in vivo* studies and translational research are needed.

### Ethics Statement

This double-blind randomized study was approved by the Kyushu University Ethical Committee under the administration of the UMIN clinical trial center (**UMIN000004803**). All participants gave written informed consent to participate after a complete description of the study.

## Supporting Information

Table S1 **Participants List.**
(XLS)

Checklist S1 **CONSORT Checklist.**
(DOC)

Protocol S1 **Trial Protocol.**
(DOC)

Protocol S2 **Japanese Version of Trial Protocol.**
(DOC)
