# Supplementary material for: Minocycline Modulates Human Social Decision-Making: Possible Impact of Microglia on Personality-Oriented Social Behaviors
Source: PLoS One. 2012 Jul 13;7(7):e40461. doi: 10.1371/journal.pone.0040461 (PMC3396661; doi:10.1371/journal.pone.0040461)
Supplement: Protocol S1 — Trial Protocol. (DOC) [file pone.0040461.s003.doc]

**Trial Protocol**

**Methods and materials**

This double-blind randomized study was approved by the Kyushu University Ethical Committee under the administration of the UMIN clinical trial center (**UMIN000004803**). Flow diagram of this study is listed on **Figure 1**. All participants gave written informed consent to participate after a complete description of the study. Participants were administered minocycline or placebo for four days, after which they played a trust game with an anonymous partner.

**Subjects**

Participants were recruited by advertisements on campus. Inclusion criterion is as follows; healthy adult males from 20 to 30 years old who can obtain informed consent. Exclusion criteria are the following five items; 1) those who ever have side effects by antibiotics including minocycline, 2) those who have severe disease in heart, liver or kidney, 3) those who have a tendency to develop allergy, and 4) those who have ever diagnosed psychiatric disorders. Their mental and physical health was confirmed by interview with a psychiatrist (TAK). All the participants were qualified for this study.

**Drug Administration**

Participants received a sheet describing their detailed dosing schedule. They were then asked to write exact time of every dosing, and to submit every package of capsules for the evidence of dosing. Participants started to take a capsule in the evening of the day and take twice a day (morning and evening) for four days afterward. On the day of the game experiment (the fifth day), they were instructed to take the last capsule three-hour prior to the appointment time for the experiment so that all of participants play the trust game under the similar drug effect. Each capsule contained 100 mg minocycline (in the treatment group) or 100 mg lactose (in the placebo group). This minocycline dose is within the range of usual daily dose used for the treatment of infections . Participants were randomly assigned to the treatment group or to the placebo group in advance, with a double-blind procedure.

**Procedure**

Prior to drug administration, participants completed a set of questionnaires (details in **Scales**). After four days of drug administration, participants were interviewed by physicians regarding side effects, other medications, and adherence to the drug administration protocol. They then played a trust game and responded to the same set of questionnaires as they completed before administration.

**Trust Game**

**Figure 2** shows the structure of trust game. In this two-player game, each player was initially given 1300 JPY (about 15 USD). The first player then decided how much of the 1300 JPY to give to the second player. The second player then went to another room, where the amount of money given to him by the first player was tripled. The second player then decided whether to split his money equally with the first player or to take all of his money. In this experiment, all the participants were actually assigned to be the first player. The first player’s decision as to how much money to give to the second player is thought to be expected the first player’s level of trust in his partner. The amount of money given was expected to be a behavioral measure of the first player’s trustfulness. In this experiment, participants had no information about the partner except that he was male. The participants thus were likely to have made their decisions based primarily on how much they trusted others in general. Participants’ partner was actually a research confederate and always the same person, a Japanese male of 22 years old. In order to control participant’s impression on the partner, the partner acted and talked exactly in the same way throughout all the experimental sessions.

**Scales**

At pre- and post-treatment, participants completed following self-rated questionnaires.

**Temperament and Character Inventory** (TCI)

TCI is based on the seven-factor model of temperament and character in personality . According to TCI model, personality is classified into temperament, which consists of Novelty Seeking (NS), Harm Avoidance (HA), Reward Dependence (RD), Persistence (PS), and character, which consists of Self-Directedness (SD), Cooperativeness (C), Self-Transcendence (ST) with a four-point Likert type scale. We used a Japanese version with 125 questions (TCI-125) , which was kindly permitted to use for this study from the HUMAN CAPITAL CONSULTING Corporation, Tokyo, Japan.

**State-Trait Anxiety Inventory (STAI)**

This anxiety scale with 20 questions consists of two factors; state anxiety, which refers to relatively unstable emotional threat to current situations, and trait anxiety, which refers to relatively stable emotional threat consistently felt in daily life .

**General Trust Scale (GTS)**

GTS consists of six questions with a seven-point Likert type scale developed by Yamagishi and Yamagishi . This scale measures respondents’ estimation of others’ trustworthiness. The reliability and validity of GTS have been confirmed across many countries . According to the past research on GTS, the major confounders of general trust are culture, sex and education level . To eliminate the effects of these confounders, we recruited homogeneous sample on sex and education level. As a result, all the participants were Japanese male and who had collage/university level education so that we could test the effect of general trust without these confounders.

**Data Analysis**

Ninety-nine Japanese males, out of 101 entries, completed our experiments (mean age 21.52 years, SD 1.65 years), and the data of them were analyzed. Among them, four participants (three in the minocycline condition, one in the control condition) failed to complete the questionnaires of STAI and GTS, so the analyses including these two scales were performed with the data of the 95 participants. All of the data analyses were performed with SPSS ver.19.
