# Supplementary material for: Minocycline Modulates Human Social Decision-Making: Possible Impact of Microglia on Personality-Oriented Social Behaviors
Source: PLoS One. 2012 Jul 13;7(7):e40461. doi: 10.1371/journal.pone.0040461 (PMC3396661; doi:10.1371/journal.pone.0040461)
Supplement: Protocol S2 — Japanese Version of Trial Protocol. (DOC) [file pone.0040461.s004.doc]

**Japanese Version of Trial Protocol**

**ミノサイクリン(minocycline)が意思決定に及ぼす影響に関する社会心理学的研究**

九州大学大学院医学研究院精神病態医学分野

九州大学先端融合医療レドックスナビ研究拠点

（早稲田大学高等研究所との共同研究）

2010年9月22日（初回承認後の条件修正版）

九州大学医系地区部局臨床研究倫理審査委員会用

(追記：2012年4月23日; Confidentialityを保つため、PLoS ONE掲載に際して、一部情報を削除改訂している)

目次

　　　　　 頁

1．研究の概要・・・・・・・・・・・・・・・・・・・・・・・・・・・・ 3

2．経緯・・・・・・・・・・・・・・・・・・・・・・・・・・・・・・・ 4

3．目的・・・・・・・・・・・・・・・・・・・・・・・・・・・・・・・ 4

4．対象　　　・・・・・・・・・・・・・・・・・・・・・・・・・・・・ 4

5．参加者の同意　・・・・・・・・・・・・・・・・・・・・・・・・・・ 5

　 6．登録方法・・・・・・・・・・・・・・・・・・・・・・・・・・・・・ 5

7．試験方法・・・・・・・・・・・・・・・・・・・・・・・・・・・・・ 6

8．注意事項　　　　　・・・・・・・・・・・・・・・・・・・・・・・・ 6

9．投与の中止および終了・・・・・・・・・・・・・・・・・・・・・・・ 7

10．目標ケース数・・・・・・・・・・・・・・・・・・・・・・・・・・・ 7

11．試験実施期間・・・・・・・・・・・・・・・・・・・・・・・・・・・ 7

12．評価項目・・・・・・・・・・・・・・・・・・・・・・・・・・・・・ 7

13．データの取扱い及び解析項目・・・・・・・・・・・・・・・・・・・　 8

14．研究発表・・・・・・・・・・・・・・・・・・・・・・・・・・・・・ 8

15．利益相反・・・・・・・・・・・・・・・・・・・・・・・・・・・・・ 8

16. 研究組織・・・・・・・・・・・・・・・・・・・・・・・・・・・・・ 9

　その他の資料

References・・・・・・・・・・・・・・・・・・・・・・・・・・・・ 10

参加者への説明文書・・・・・・・・・・・・・・・・・・・・・・ 　　　　12

同意書・・・・・・・・・・・・・・・・・・・・・・・・・・・・・　　 17

症例登録確認表・・・・・・・・・・・・・・・・・・・・・・・・・　　 18

募集広告・・・・・・・・・・・・・・・・・・・・・・・・・・・　　 20

# 1．研究の概要

１）スタディータイプ：精神科医師・社会心理学者との共同自主研究

２）目的：ミノサイクリンを健常者に投与し、その際の認知および社会的行動の変化を社会心理学実験により調べる。

３）対象：20歳以上の成人男子で下記の条件を満たすこと

　　　(1)男子学生

　　　(2) 年齢２０歳以上

　　　(3) 文書による事前同意が得られる者

除外基準

　　　(1) 過去にミノサイクリンを含む抗生剤過敏症の既往歴のある者。

　　　(2) 高度の心・肝・腎障害をもつ者

　　　(3) アレルギ－症状の体質を有する者。

　　　(4) 精神疾患をもつ者（既往を含む）。

　　　(5) その他、試験担当医師が不適当と判断した者。

４）研究計画

　本試験は、無作為対照化試験である。対象者を100名選抜し、はじめに、ランダム（無作為）に２つの投薬群に分ける。（本物のミノサイクリンが投与される群または、プラセボ薬が投与される群）に割付する（詳細は、７の①を参照）。

試験開始日に問診及び心理評価の質問紙（社会に関する考え方や対人関係についての質問紙）に記載してもらう。同日より、4日間ミノサイクリン100mgカプセルを朝夕食後二回内服してもらう。4日間の投薬後、再び同様の質問紙に回答してもらい、金銭を使った取引の社会心理実験（信頼テスト・資源分配テスト）に参加してもらう。この取引の実験で得た金額は、実験参加による謝金とは別に支払われる。

５) 評価項目

心理評価の質問紙

信頼テスト

など

６）予定対象者の数100例

７）試験期間：平成22年10月中旬（予備期間：12月中旬）に実施予定

８）問合せ先　　試験責任医師；教授　神庭重信

試験分担医師；講師・門司晃、特任助教・加藤隆弘、大学院生・堀川英喜

実験担当研究者；早稲田大学高等研究所　准教授・渡部幹

連絡先　九州大学大学院医学研究院精神病態医学分野　加藤隆弘

：０９２－６４２－＃＃＃＃　精神科研究棟（平日8:30～17:00）

：０９２－６４２－＃＃＃＃　精神科病棟（夜間休日）

**２．経緯**

　　近年社会心理学的問題に対する脳科学のアプローチが盛んになっており、その中でも代表的なものは脳内ホルモンであるオキシトシンが人々の間の信頼に深くかかわっているという知見(Kosfeld et al. 2005)である。この例に代表される一連の研究では、脳神経細胞に直接働きかける物質を対象としている。これに対し、本研究は脳神経細胞ばかりではなく、脳神経細胞の働きを制御する脳内免疫細胞ミクログリアに着目し、ミクログリア活性を抑制する薬物であるミノサイクリンを用いて、上記の社会的ストレスの問題にアプローチしようというものである。

ミノサイクリンは代表的な第三世代抗生物質で、細菌性肺炎から慢性皮膚感染症に至るまで幅広く用いられる安全性の確立した薬物である。近年、ミノサイクリンは抗菌作用以外に抗炎症作用を有することが判明し、脳内では免疫担当細胞であるミクログリアを介した抗炎症機序による脳保護作用が示唆され、認知症・多発性硬化症などの神経変性疾患への治療効果が期待されている。

とりわけ、統合失調症患者がミノサイクリンを内服すると精神症状が改善したという報告(Miyaoka et al. 2007; Miyaoka et al. 2008)などから、ミノサイクリンは統合失調症やうつ病などの精神疾患の治療薬としての可能性も示唆されている。心理・精神活動と、脳内免疫細胞であるミクログリアとの相関は全く未知であるが、これらの報告などから、ミクログリア活性化が病的な心理・精神活動に重要な貢献をしている可能性が仮定される。齧歯類を用いた動物実験で、強いストレス下にミクログリアの活性化を認めるという最近の報告(Sugama 2009; Sugama et al. 2007; Sugama et al. 2009)を鑑みると、精神疾患の症状に限らず、不安・恐怖などの原始的感情の制御と向社会的行動の発現にミクログリアが関与している可能性がある。

以上の知見を総合すると、ミノサイクリン投薬による健常者の不安や恐怖といった感情の変化と、信頼感や公正感といった社会心理学的に重要な心理機能の変化を測定することが未知のミクログリアの精神機能への関与を検討する重要な礎になることが期待される。したがって、本研究では、健常者に対してミノサイクリンを投与し、不安尺度（STAI）、簡易版Temperament and Character Inventory (TCI)、一般的信頼尺度（他者一般に対する信頼度）などの変化を測定するほか、社会心理学的な行動指標として信頼ゲームや独裁ゲームと呼ばれる行動経済学で用いられる対人的資源取引を行ってもらい、統制群（対照群）と比較する。そして、これらの結果を総合的に分析することで、精神疾患予防として、また向社会的行動促進の重要因子としてミクログリアの働きについて、より詳しい知見と新たな仮説を導くとともに、精神疾患予備群である健常者に対する精神衛生のための方策について新たな示唆を得ることを目的とする。

**３．この研究によって実証しようとする仮説（目的）**

ミノサイクリンの投与によって、人（健常者）の対人不安が減少し、他者への信頼、愛他性が高まり、向社会的行動が促進されるという仮説を検証する。

**４．対象**

　　1)参加対象者

　　　(1)男子学生

　　　(2) 年齢２０歳以上

　　　(3) 文書による事前同意が得られる者

3)除外基準

　　　(1) 過去にミノサイクリンを含む抗生剤過敏症の既往歴のある者。

　　　(2) 高度の心・肝・腎障害（Cr>1.5）の者

　　　(3) アレルギ－症状の体質を有する者。

　　　(4) 精神疾患をもつ者（既往を含む）。

　　　(5) その他、試験担当医師が不適当と判断した者。

**５．被験者の同意**

本実験実施に先立ち、試験担当医師は対象者に対し、下記事項を別紙説明文書に基づいて事前に十分説明し、原則として２部文書にて参加者の自由意志による同意を取得する。同意が得られた場合には１部は精神病態医学分野（臨床研究棟1階）内の鍵のかかるロッカーに保存し、他の１部は参加者が保管し、試験終了後も回収しない。なお、止むを得ず口頭により同意を得た場合には、その理由を含め口頭同意記録を残すものとする。

1. 実験の概要
2. 実験の目的
3. 実験方法
4. この実験の予想される効果と、起こるかもしれない副作用及び不利益について

　　　(7)健康被害が発生した場合の補償について

　　　(8)実験への参加とその撤回について

　　　(9)実験を中止する場合について

　　 (10)この実験に関する情報提供について

(11)プライバシーの保護について

(12)費用について

(13)利益相反について

(14)この実験を担当する医師及び健康被害が発生した場合の連絡先

**６．登録方法**

1)登録手順

　事前に同意書が取れている対象参加者が適格基準をすべて満たし、除外基準の何れにも該当しないことを再確認し、症例登録用紙に必要事項をすべて記入の上、試験分担医師でデータ管理を担当する加藤隆弘が、登録データを鍵のかかるロッカーに保管する。

被験者登録の連絡先　　精神科医局：TEL：092-642-####

　　 FAX：092-642-####

2)登録に際しての注意事項

　1.プロトコール実験開始後の登録は例外なく許容されない。

　2.症例登録用紙の記載が不十分な時は、すべて満たされるまで登録は受付けられない。

　3.試験分担医師・加藤隆弘により適格性が確認された後に、登録番号が発行される。

　4.登録されると登録確認通知が精神科医局より、実験担当者に送信する。

　5.一度登録された被験者は登録取消し（データベースから抹消）はなされない。重複登録の場合はいかなる場合も初回の登録情報(登録番号)を採用する。

　6.誤登録・重複登録の場合が判明した際には、すみやかに試験分担医師・加藤隆弘に連絡すること。

**７．研究の実施手順**

研究手順は①被験者プールの作成、②被験者に対する投薬前実験、③被験者への投薬、④被験者に対する投薬後実験の４つの段階に分けられる。以下に各段階について述べる。

1. 被験者プールの作成

九州大学のポータルサイトにて100人の被験者（男子のみ100名、20歳以上）を募る。その際、１）健康上大きな問題がないこと、2) ミノサイクリンを含む抗生物質等の服薬によりこれまでアレルギー等の症状発現がないことを事前に尋ねておき、これらの条件を満たすものだけをリクルートする。ランダム化に関しては、被験者データを管理し直接被験者と遭遇することがない試験分担医師・加藤隆弘が行う。方法としては、乱数表を用いて、投薬群、プラセボ群（コントロール群）に分ける。九州大学病院の外部に個人情報が漏れないように、早稲田大学の協力を得て行う実験に関しては、被験者が同定される個人名を用いずに、エントリー時のID番号のみを外部に公開することで匿名化をはかる。

1. 投薬前実験

投薬を行う前に、下記12の４つの自記式調査票に回答してもらう。

1. 投薬

精神保健指定医でかつ精神科専門医である担当医師が被験者それぞれを問診し、身体状態と精神状態に医学的問題のないことを確認したうえで、ミノサイクリンを処方する。この際、半数の被験者にはプラセボ薬を処方する。ミノサイクリンとプラセボ薬（外観はミノマイシン錠と類似）に関しては、アステム株式会社から直接購入する。被験者には、薬と偽薬の可能性があること説明し、その同意を得たうえで処方する。ミノサイクリンとプラセボ薬は錠剤で処方し、1日2回、4日間服用してもらう。飲み忘れ等を防ぐため、被験者に十分に説明を行い、実験終了後に検尿・採血にて服薬コンプライアンスの確認を行う可能性があることを告げておく。

1. 投薬後実験

投薬前と同様の自記式調査票に回答してもらい、その後行動実験を行う。この実験は九州大学馬出キャンパス内・コラボステーションI 3階・心理実験室（九州大学大学院人間環境学府・橋彌和秀准教授のラボ）を使って行われる予定で、実験実施は渡部（早稲田大学）が担当する。

**８．実験期間中の注意事項**

原則として、実験期間中は、過度な運動や飲酒を避けること。

**９．投与の中止および終了**

　　次の項目のいずれかに該当する場合、試験担当医師の判断で中止する。この場合は投与中止時点で所定の検査を実施し、中止・終了の年月日、理由および中止後の経過・処置について調査票に記載する。

　　　　(1)副作用または臨床検査値異常が発現し、継続投与が困難と判断された場合。

　　　　(3)対象から除外すべき条件に該当することが、投与開始後に判明した場合

　　　　(4)その他、試験担当医師が継続投与を不適当と判断した場合。

　　　随伴症状および臨床検査値異常変動が発現した場合には、その症状、発現日、

　　　　程度、処置、経過、および薬剤との因果関係などについて詳細に記録する。

また、本試験でみられた有害事象のうち、本剤との因果関係を明らかに否定できない症例を副作用とし、副作用発現率を評価する。

　　なお、IRBの承認がおりた際には、日本興亜損保と賠償契約を締結し、副作用に関する医療費等は賠償保険で補償いたします。

**１０．目標ケース数**

男子学生100例

**１１．試験実施期間**

平成22年10月12日～平成22年12月15日に実施予定（平成22年度中に完了する）。

**１２．評価項目**

実験開始日及び最終日に、主に、以下の４つの自記式調査票に回答してもらう。

1. 不安尺度（STAI）
2. 簡易版Temperament and Character Inventory (TCI)
3. 一般的信頼尺度
4. 公正自己尺度

その他

最初の２つの尺度はパーソナリティ尺度であり、臨床実験において最も一般的に用いられているものである。残りの2つは社会心理学で用いられているもので、対人相互作用の行動予測に用いられている。

さらに、「信頼ゲーム」と呼ばれる資源取引の実験を行う。これは社会心理学や行動経済学で良く用いられるものであり、金銭を他者に預けることで、より多くの利益を得る可能性がある一方、損益を出す可能性もある取引である。この取引状況では、その他者の善良性をどの程度信用できるかによって預ける額が変わることが多くの先行研究で知られているため、このゲームを用いて、他者信頼の行動的指標を取る。またもうひとつ、見知らぬ他者とペアを組み、そのペアの代表として自分が預けられた金額の分配配分を決定する「独裁ゲーム（資源分配ゲームの一つ）」も被験者に行ってもらう。このゲームはどれだけ「公平な」資源分配を志向するかを測定するための行動指標を提供する。

この実験の目的は、私たちの現実の社会関係と同様に、他者の行動によって自分の利得の全部ないし一部が決定される関係において、人々がお互いの利害調整をどのように行うかを調べることにある。この目的のためには、実験謝礼金額そのものが被験者の決定の組み合わせによって変動するような実験を行う必要があり、上記のゲーム状況は社会心理学や実験経済学で最も頻繁に用いられているものであり、NatureやScience誌をはじめとする主要学術誌にもこれらのゲームを用いた実験結果は多く掲載されている。

以上のように質問紙法とゲーム実験による行動指標という2種類の方法を使って、仮説検証を行うこととする。

**１３．データの取扱い及び解析項目**

得られたデータは、氏名と対応する番号で整理し、一括して試験分担医師・加藤隆弘が、登録データを鍵のかかるロッカーに保管する。統計解析に関しては、SPSS（日本語版）を用いて、信頼感尺度などの尺度スコアを投薬前後および投薬群と偽薬群で比較し、その変化に統計的な有意差があるかどうかを検定する。さらに、投薬後の信頼ゲームと最後通牒（独裁）ゲームでの意思決定結果（金銭取引実験のため金額が指標となる）を投薬群と偽薬群とを比較し、ミノサイクリンが行動に与える影響に有意な差があるかを検定する。さらに尺度スコアとゲームでの意思決定金額との関係を相関分析、回帰分析などを用いて解析する。

**１４．研究発表**

結果の如何に関わらず、学会発表及び論文発表を行う。

**１５．利益相反**

本試験において、ミノマイシンの製造元・発売元のワイス株式会社・武田薬品工業株式会社・アステム株式会社を含むあらゆる団体・企業との間で利益相反はない。

**１６．研究組織**

九州大学大学院精神病態医学分野において、本臨床試験に賛同いただいた施設を対象に実施いたします。

研究代表者:　教授　神庭重信

試験分担医師:講師　門司晃、特任助教　加藤隆弘、大学院生　堀川英喜

九州大学大学院精神病態医学分野

〒812-8582 福岡市東区馬出3-1-1

電話092-642-5627　(夜間・休日)092-642-####

他施設・分担研究者:　 早稲田大学高等研究所　准教授　渡部　幹

研究事務局

九州大学大学院精神病態医学分野

登録事務局

九州大学大学院精神病態医学分野　加藤隆弘

〒812-8582　福岡市東区馬出3-1-1

電話092-642-####　　FAX092-642-####

# References

Bian Q, Kato T, Monji A, Hashioka S, Mizoguchi Y, Horikawa H, Kanba S. 2008. The effect of atypical antipsychotics, perospirone, ziprasidone and quetiapine on microglial activation induced by interferon-gamma. Prog Neuropsychopharmacol Biol Psychiatry 32(1):42-8.

Crockett MJ, Clark L, Tabibnia G, Lieberman MD, Robbins TW. 2008. Serotonin modulates behavioral reactions to unfairness. Science 320(5884):1739.

Kato T, Mizoguchi Y, Monji A, Horikawa H, Suzuki SO, Seki Y, Iwaki T, Hashioka S, Kanba S. 2008. Inhibitory effects of aripiprazole on interferon-gamma-induced microglial activation via intracellular Ca2+ regulation in vitro. J Neurochem 106(2):815-25.

Kato T, Monji A, Hashioka S, Kanba S. 2007. Risperidone significantly inhibits interferon-gamma-induced microglial activation in vitro. Schizophr Res 92(1-3):108-15.

Kosfeld M, Heinrichs M, Zak PJ, Fischbacher U, Fehr E. 2005. Oxytocin increases trust in humans. Nature 435(7042):673-6.

Miller G. 2005. Neuroscience. The dark side of glia. Science 308(5723):778-81.

Miyaoka T, Yasukawa R, Yasuda H, Hayashida M, Inagaki T, Horiguchi J. 2007. Possible antipsychotic effects of minocycline in patients with schizophrenia. Prog Neuropsychopharmacol Biol Psychiatry 31(1):304-7.

Miyaoka T, Yasukawa R, Yasuda H, Hayashida M, Inagaki T, Horiguchi J. 2008. Minocycline as adjunctive therapy for schizophrenia: an open-label study. Clin Neuropharmacol 31(5):287-92.

Monji A, Kato T, Kanba S. 2009. Cytokines and schizophrenia: Microglia hypothesis of schizophrenia. Psychiatry Clin Neurosci 63(3):257-65.

Steiner J, Bielau H, Brisch R, Danos P, Ullrich O, Mawrin C, Bernstein HG, Bogerts B. 2008. Immunological aspects in the neurobiology of suicide: elevated microglial density in schizophrenia and depression is associated with suicide. J Psychiatr Res 42(2):151-7.

Steiner J, Mawrin C, Ziegeler A, Bielau H, Ullrich O, Bernstein HG, Bogerts B. 2006. Distribution of HLA-DR-positive microglia in schizophrenia reflects impaired cerebral lateralization. Acta Neuropathol 112(3):305-16.

Sugama S. 2009. Stress-induced microglial activation may facilitate the progression of neurodegenerative disorders. Med Hypotheses. In press

Sugama S, Fujita M, Hashimoto M, Conti B. 2007. Stress induced morphological microglial activation in the rodent brain: involvement of interleukin-18. Neuroscience 146(3):1388-99.

Sugama S, Takenouchi T, Fujita M, Conti B, Hashimoto M. 2009. Differential microglial activation between acute stress and lipopolysaccharide treatment. J Neuroimmunol 207(1-2):24-31.

van Berckel BN, Bossong MG, Boellaard R, Kloet R, Schuitemaker A, Caspers E, Luurtsema G, Windhorst AD, Cahn W, Lammertsma AA and others. 2008. Microglia activation in recent-onset schizophrenia: a quantitative (R)-[11C]PK11195 positron emission tomography study. Biol Psychiatry 64(9):820-2.

**説 明 文 書**

「ミノサイクリン(minocycline)が意思決定に及ぼす影響に関する社会心理学的研究」のご説明

ミノサイクリン(minocycline)が意思決定に及ぼす影響に関する社会心理学的研究

　この説明文書は、あなたにミノサイクリン（minocycline＝商標名ミノマイシン（ワイス製薬））投与実験の内容を正しく理解していただき、あなたの自由な意思にもとづいて、この試験に参加するかどうかを判断していただくためのものです。この説明文書をお読みになり、担当医からの説明を聞かれた後、十分に考えてからこの試験に参加するかどうかを決めて下さい。たとえ参加されなくても、今後の学業や学生生活に不利益になることはありません。また、不明な点があればどんなことでも気軽に質問して下さい。

1. **試験について**

本研究はミノサイクリンを健常者に投与し、その際の認知および社会的行動の変化を調べることを目的としています。ミノサイクリンは呼吸器感染症から皮膚感染症に至る幅広い感染症に用いられる安全性の確立した抗生物質で、近年新たに脳保護作用が判明し、うつ病や統合失調症など精神疾患の治療薬としても期待されています。最近の基礎研究では、ミノサイクリンは脳内免疫細胞ミクログリアの制御を介して脳保護的に機能することが判明しています。ミクログリアの精神機能への関与は未知ですが、活性化ミクログリアは、不安を高めたり、興奮を誘導する様々な神経伝達物質を産生することが判明しています。したがって、本研究においてミノサイクリンを健常者に処方した場合、極度の不安も極度の興奮も抑制するという、これまでになく新しい情動安定化効果が期待されます。なお、ミノサイクリンについての精神心理学領域での人を用いた研究は、認知症・統合失調症・自閉症など精神疾患患者に限られていますが、健常者への安全性は　30年以上抗生物質として幅広く処方されていることから、すでに確立されています。青年期においても、挫創（にきび）の治療薬として有名で、挫創治療の第一選択薬として安全性の高い薬剤として処方されています。従って、本研究では、健常者への処方も差し支えないと判断し、健常者への投薬の実施と、その前後での向社会性についての心理学的データを収集します。

1. **あなたの病気について**

　先に書かれていたように、この薬を用いた実験は、特に重い病気を持たず、日常生活を支障なく送ることのできる方々を対象として行います。

　したがって、ミノサイクリン投与が危険となるような、何らかの病気や障害をお持ちでなければ、この臨床試験を受けることができます。くすりの投与後、まれに副作用がでることがあります。副作用の種類とそれに対する処置については、この後に書かれていますので、必ずご参照ください。

**３．あなたの病気に対する治療法について**

先に書かれていたように、この実験は、特定の病気の治療を目的としたものではありません。

**４．試験の目的**

　この試験の目的は、健常者に対して、おくすり（ミノサイクリン）を処方した場合、社会に対する価値観や対人関係の見方、そして対人的意思決定がどのようなに変化するかを調べることです。

**５．試験の方法**

　本試験は、九州大学構内に告知された掲示板をご覧になり、この研究に興味を持った九州大学の男子学生さん約100名を対象に行われます。実験希望者にはまず、医師による健康診断を受けていただき、おくすりの投与について問題がないことを確認します。その際、腎臓や肝臓に病気のある方、その時の健康状態の悪い方（風邪など）、また過去にミノサイクリン、あるいは、類似のおくすりの投与によって副作用の出た方は、実験の対象にはなりません。

　本試験では、同意いただいた方たちを、ランダム（無作為）に２つの投薬群に分けます。（本当のミノサイクリン投与される群または、プラセボ薬が投与される群）に割付します。計画では、それぞれの群で約50名ずつが登録される予定となっています。どちらの群に割り振られるかは、コンピューターによってランダムに決定され、あなたはどちらのグループに割り振られるかを選択することはできません。

試験開始日から、参加者の方には毎日所定の実験室にお越しいただき、医師によって健康状態の変化と副作用について健診を受け、問題ない場合には一日分のおくすりが渡されます。おくすりは100mgのカプセル錠で一日2回、朝夕食後にお飲みいただきます。これを4日間続けていいただきます。

　試験開始日、おくすりを飲んでいただく前に、社会に関する考え方や対人関係についての質問紙（80問程度）にお答えいただきます。4日間の投薬後、再び同様の質問紙にお答えいただく他、金銭を使った取引の実験に参加していただきます。この取引の実験で得た金額は、服薬実験参加による謝金（####円）とは別に支払われます。4日目に服薬状況を把握するために検尿・採血をすることがあります。

　試験の具体的なデザイン（治療と検査の計画）を以下に示します。

| 「試験開始日－4日目」　　　　　　　　「試験5日目」  本当のお薬を飲む群  「試験開始日」  質問紙に回答　　　　　　　　　　　　　　　　　　　　　　　　　　　　　　質問紙に回答  医師による  健康診断　　　　　　　　　　　　　　　　　　　　　　　　　　　　　　　　取引実験に参加　　　　終了    　　　　　　　　　　　　　　　　　　　偽のお薬を飲む群 |
| --- |

**5.1金銭を使った取引実験について**

　　投薬後の金銭を使った取引実験は、以下の通りです。

　　実験参加者は他の実験参加者の誰か一人とペアになります。ただし、ペアリングは実験者が行い、参加された皆さんは誰と組んでいるかはわからないようになっています。実験中も実験後も誰と組んだのかはわかりませんし、それは皆さんの相手にとっても同様です。さらに実際に実験を行う実験者にも皆さんがどのような行動をとったかがわからないように工夫されています。

　　皆さんには2種類の取引を行っていただきます。一つは「投資取引」と呼ばれるものです。ペアのそれぞれには取引の最初に元手として一定の金額（1300円）ずつが渡されます。その後ペアは第一プレイヤーと第二プレイヤーに割り当てられます。割り当ては実験者によってランダムに決まります。取引では、まず、第一プレイヤーが元手の1300円のうち、好きな額を相手に預けるという決定をします。次に、預けられた第二プレイヤーは、別室に行き、その金額を増やすことができます。そして、増えた金額をすべて自分のものにするか、預けた相手と平等に分けるかを選択します。皆さんには相手にいくら預けるか、また預けられた場合にはどうするかを決定していただきますこの実験にかかる時間はおよそ30分ほどです。この実験での報酬額は理論的には0円から3900円となりますが、0円にならないように自分で決定することができます。また実験を途中で棄権する権利が認められています。

**６．この試験薬の予想される効果と、起こるかもしれない副作用および不利益について**

　ミノサイクリンは呼吸器感染症から皮膚感染症に至る幅広い感染症に用いられる安全性の確立した抗生物質ですが、以下のような副作用が報告されています（詳細は、**別紙副作用リスト**を参照ください）。主な副作用としては、腹痛（３．０７％）、悪心（３．０４％）、食欲不振（１．８８％）、胃腸障害（１．１３％）等の消化器症状、眩暈感（２．８５％）などが報告されています。その他にけいれんやアレルギー肝障害等の副作用の発現がまれにみられることも報告されています。稀に起こる重篤な副作用として、ショック、アナフィラキシー様症状、Stevens-Johnson症候群が報告されています。

　もし、好ましくないと思われる症状が現れましたら、直ちに適切な処置を施しますので、どんな事でも担当医師まで申し出てください。また、重大な合併症が生じた際にはこの試験の中止も含め、病院長をはじめ担当部署に報告し協議いたします。

**７．健康被害が発生した場合について**

この実験は慎重に行いますが、実験の期間中、あるいは終了後にあなたに副作用などの健康被害が生じた場合には、医師が適切な診察と治療を行います。本研究は日本興亜損保と賠償契約を締結しております。したがって、万が一副作用が発現した際の治療や検査等の費用については賠償保険で補償されます。

**８．試験への参加とその撤回について**

　この実験に参加されるかどうかはあなたご自身の自由意思によります。これを拒否されてもそのことにより不利益を受けることはありません。また同意後治療の開始の有無に関わらず、いつでも撤回できます。あなたがこの試験に参加される事に決めた後でも、あなた自身の都合でいつでも辞退することができます。

**９．試験を中止する場合について**

　あなたが試験の中止を希望した場合、副作用のために担当医が試験を中止した方がよいと判断した場合、試験の中止規定に相当した場合（風邪をひいた、体調を崩した、などの症状があり、医師が中止を判断した場合、おくすりを指示通りに服用できなかった場合）は試験を中止します。試験中止後も、その後の対応について担当医師が誠意をもって相談に応じます。

**１０．この試験に関する情報は、随時ご連絡いたします。**

この試験に関して、参加の継続についてあなたのご意思に影響を与える可能性のある情報が得られた場合にはすみやかにお伝えします。

**１１．プライバシーの保護について**

　この臨床試験の結果は学会発表や論文での報告などに使用しますが、あなたご自身のプライバシーに関する秘密は全て厳守します。名前や個人を識別する情報は、報告に当たって一切使用しません。

**１２．費用について**

この研究に参加するにあたり、あなたが支払うべき費用が生じることは、ありません。しかし、例外として、副作用が生じた場合に、その治療や検査等の費用については、通常の診療と同様にみなさんの健康保険を用いて対処する事となります。

**１３．利益相反について**

　試験に使用する医薬品「ミノマイシン」は、ワイス株式会社・武田製薬株式会社が製造・販売し、アステム株式会社が卸販売したものを使用します。また、九州大学は、今回の研究に際して、上記の各社から試薬提供及び寄付等は一切受けておりません。

**１４．試験を担当する医師および健康被害が発生した場合の連絡先**

この試験のことで何かわからないことや心配なことがありましたら、いつでも、ここに記載されている医師または相談窓口にお尋ねください。

九州大学大学院精神病態医学分野　試験責任医師：教授　神庭重信

試験分担医師(連絡受付け担当)：講師 門司晃,特任助教 加藤隆弘,大学院生 堀川英喜

　　　　　連絡先：　　　TEL : 092-642-####　精神科研究棟（平日8:30～17:00）

　　　　　　　　夜間 ：092-642-#### 精神科病棟　（夜間休日）
